# Comment on Howe’s Root Filling

**Published:** 1919-02

**Authors:** U. G. Rickert


### COMMENT ON HOWE'S ROOT FILLING.

BY U. G. RICKERT, D.D.S.

I can think of no greater service to the dental profession today than the standardization of root canal operations. Personally, I would have preferred to have been excused from offering any data at this time because of the limited amount of work I have as yet done in this field. On the other hand, I realize that if all of us waited until the last word had been said on the subject, much valuable time might be lost.

Inasmuch as I had never intended to formally report the work that I have done, I will be obliged to give a recital of experience rather than a concise tabulation of facts developed by special research. I mention this because it will explain this report.

Early last fall I planned to work out a practical routine for root canal treatment for both my private practice and for clinical purposes. I decided to make cultural tests to determine sterility of all canals before filling, and I was certainly surprised to find the utter inefficiency of one drug after another in which I had confidence. I found that one of my pets, phenol-compound, which I had been using in anterior teeth, could not be relied upon for sterilizing badly infected canals. Most dressings were infected in twenty-four hours and practically all in forty-eight hours. I had been depending on iodized-phenol in posterior teeth but found it likewise undependable. Formocresol, while maintaining sterility longer than either of the above, also became infected after several days to a week. Oil of cloves is of no value as a disinfectant.

As an experiment I took single rooted teeth, fastening them into thin cork so that two-thirds of the root passed through the cork. These are then fitted into short glass tubes filled with media, after which they are autoclaved. I took several of these and reduced silver into the canals and left them standing for twenty-four hours, after which I sealed into the pulp chamber a mixed culture of the flora of my own mouth, and in no instance was there any growth in the media. I had sealed the apical foramina. Experiments with silver, a modification of Howe's method, is the only way I have been able to sterilize canals with certainty. There are serious objections to this method, however, owing to the possible irritation, if thorough sterilization of the root canals to the apex is to be had. It is somewhat difficult to control the exact amount to be administered in each case and avoid destruction of periapical tissue. I have, however, modified Dr. Howe's method so that it is less irritating, by using a minimum amount of water with a maximum of silver. This may be done by using the strong ammonia and dropping in silver nitrate until there is but slight, if any, precipitation of silver oxide in solution of ammonia. This solution should be freshly prepared before use. Care in administration will prevent serious irritation.

Another precaution that must be considered is the purity of drugs. Observation by myself and others has convinced us that some of the serious irritations that have followed the use of this treatment have been due to impurities, especially of the ammonia. Another objection to this method which limits its use is that of the dark discoloration.

I am at present working on a method intended to remove the serious objections of the silver method. This is not definite enough yet to be given out. I mention the silver method because laboratory tests have convinced me of its efficiency. It will sterilize dentin to the cementum.

I also found by using the tubes described above with mixed cultures in the media, that dressings sealed into canals were unable to maintain sterility for any length of time.

As will be observed, the agents tested were not an extensive list, but the ones we had been accustomed to use and depend upon. The small amount of work I put into it certainly convinced me of the unreliability of most of the agents we are now using. Let us hope that something may soon be found which will be effective with the least injury to the periapical area. I shall await with interest the data to be collected for the Chicago meeting.—Extract from Dr. Hartzel's report in *Journal N. D. A.*, January, 1919.

